# Differential Regulation of Cutaneous Oncoprotein HPVE6 by wtp53, Mutant p53R248W and ΔNp63α is HPV Type Dependent

**DOI:** 10.1371/journal.pone.0035540

**Published:** 2012-04-18

**Authors:** Jian-Wei Fei, Ethel-Michele de Villiers

**Affiliations:** Division for the Characterization of Tumorviruses, Deutsche Krebsforschungszentrum, Heidelberg, Germany; IPO, Inst Port Oncology, Portugal

## Abstract

UV exposure and p53 mutations are major factors in non-melanoma skin cancer, whereas a role for HPV infections has not been defined. Previous data demonstrated the wtp53-mediated degradation of cutaneous HPV20E6 by caspase-3. ΔNp63α and hot-spot mutant p53R248W conveyed a protective effect on HPV20E6 under these conditions. We demonstrate a differential regulation by wtp53 of the E6 genes of cutaneous types HPV4, HPV5, HPV7, HPV27, HPV38, HPV48, HPV60 and HPV77. Caspase- or proteasome-mediated down-regulation was HPV type dependent. Mutant p53R248W up-regulated expression of all these E6 proteins as did ΔNp63α except for HPV38E6 which was down-regulated by the latter. None of these cellular proteins affected HPV41E6 expression. Ectopic expression of both mutp53R248W and ΔNp63α in the normal NIKS keratinocyte cell line harbouring endogenous p53 and p63however led to a down-regulation of HPV20E6. We demonstrate that HPV20E6 expression in these cells is modulated by additional, yet unidentified, cellular protein(s), which are not necessarily involved in apoptosis or autophagy. We further demonstrate proliferation of HPV20E6-expressing keratinocytes. Levels of proteins involved in cell cycle control, cyclin-D1, cdk6 and p16^INK4a^, phosphorylated pRB, as well as c-Jun and p-c-Jun, were all increased in these cells. HPV20E6 did not compete for the interaction between p16^INK4a^ with cyclin-D1 or cdk6. Phosphorylation of pRB in the HPV20E6 expressing cells seems to be sufficient to override the cytokenetic block induced by the p16^INK4a^/pRB pathway. The present study demonstrates the diverse influence of p53 family members on individual cutaneous HPVE6 proteins. HPV20E6 expression also resulted in varying protein levels of factors involved in proliferation and differentiation.

## Introduction

Cutaneous papillomaviruses (HPV) have been associated with the pathogenesis of non-melanoma skin cancer. The wide spectrum of HPV types demonstrated by DNA detection in malignant lesions also occurs in normal skin [1]–[8]. The mechanism by which these viruses contribute to malignant disease remains unclear. A crucial function of high-risk mucosal HPV E6 in the pathogenesis of malignant tumors is targeting a number of cellular proteins, including wtp53, for proteasomal degradation [9]–[12]. Cutaneous HPVs do not induce proteasome-mediated degradation of p53 or PDZ-domain proteins [11], [13], [14]. The majority of so-called cutaneous HPV types belong phylogenetically to the genera Beta- and Gamma-papillomaviruses, although a few types which are mainly associated with benign lesions of the skin, group within the genus Alpha-papillomavirus [15], [16]. Evidence on the molecular activity of single cutaneous HPV types is slowly emerging. Recent results indicate that the activation of telomerase by HPV38E6 may prolong the lifespan of human keratinocytes [17], [18]. A number of cutaneous HPV types, in contrast to others, have transforming potential in rodent cells [19], [20].

UV-exposure and mutations in wtp53 are considered as co-factors in the pathogenesis of non-melanoma skin cancer [21], [22]. A number of p53 mutations have been termed “hot-spot" mutations due to their frequent association with respective tumor types [23]. p53 mutantR248W is a UV-induced “hot-spot" mutation in non-melanoma skin cancer. Mutant p53 binds to promoters to form transcriptionally active complexes, thereby gaining function [24], [25]. The contact-mutant p53R248W exerts a dominant-negative effect through tetramerization with wtp53 and other p53 family members, with re-localization of this complex to the nucleus [26].

TAp63α and ΔNp63α play an important role in proliferation and differentiation of the skin and the ratio between these two isoforms determines the biological outcome. Increased level of ΔNp63αleads to failure of differentiation and the organization of the epithelium [27]. Proliferation and differentiation defects in the skin of p63-null mice were rescued by the direct down-regulation of p16^INK4a^ expression by p63 [28]. ΔNp63α acts as a dominant negative by inhibiting p53, TAp63 and TAp73 trans-activation and thus apoptosis [29], [30] and is over-expressed in several tumors including the majority of squamous cell carcinomas [31]–[33].

E6 gene expression of several cutaneous HPV types protected keratinocytes from UV-B induced apoptosis [34]–[36] by mediating degradation [34] or a reduction in the levels of pro-apoptotic Bak [37] and thereby preventing the release of pro-apoptotic factors from mitochondria [38]. We demonstrated that p53-mediated caspase-dependent degradation of HPV20E6 was rescued by mutant p53R248W and ΔNp63α and other unknown proteins involved in proteasome degradation [13]. We now investigated whether similar interactions between mutant p53R248W, wtp53 and ΔNp63α with E6 of other cutaneous HPV types occurred, as we had previously demonstrated a type specific, rather than genus- or species-specific UV-induced activation or suppression of a number of cutaneous HPV promoters [36], [39]. The present study demonstrates a differential regulation of the E6 genes of cutaneous types HPV4, HPV5, HPV7, HPV27, HPV38, HPV48, HPV60 and HPV77 by wtp53. Caspase- or proteasome-mediated down-regulation was HPV type dependent. The majority of these E6 proteins were upregulated by both mutant p53R248W and ΔNp63α. An exception was HPV38E6 which was down-regulated by ΔNp63α. None of these cellular proteins affected the expression of HPV41E6. These data were obtained by over-expression in the p53-null H1299 cell line. We continued to examine whether similar observations could be made in human keratinocytes with endogenous wtp53 and p63. We limited these further studies to the expression of HPV20E6 in human keratinocytes. Ectopic expression of both mutp53R248W and ΔNp63α in the normal NIKS keratinocyte cell line harbouring endogenous p53 and p63 however led to a down-regulation of HPV20E6. We demonstrate that HPV20E6 expression in these cells is modulated by additional, yet unidentified, cellular protein(s) which are not necessarily involved in apoptosis or autophagy. We further demonstrate proliferation of HPV20E6-expressing keratinocytes. Levels of proteins involved in cell cycle control, cyclin-D1, cdk6 and p16^INK4a^, phosphorylated pRB, as well as c-Jun and p-c-Jun, were all increased in these cells. HPV20E6 did not compete for the interaction between p16^INK4a^ with cyclin-D1 or cdk6. Phosphorylation of pRB in the HPV20E6 expressing cells seems to be sufficient to override the cytokenetic block induced by the p16^INK4a^/pRB pathway.

## Materials and Methods

### Expression vectors

Plasmid constructs pcDNA3.1(+)-mtp53R248W, pcDNA3.1(+)-wtp53 and pcDNA3.1(+)-ΔNp63α were described previously [40]. N- and C-terminal flag-tagged HPV4E6, HPV5E6, HPV7E6, HPV20E6, HPV27E6, HPV38E6, HPV41E6, HPV48E6, HPV60E6 and HPV77E6 were obtained by PCR amplification using the respective complete genome as template (primers in Table S1) [13], [41]. All amplified products were cloned into vector pcDNA3.1(+) from Invitrogen (Karlsruhe, Germany). Sequences of all DNA inserts and their orientation were verified by sequencing of the constructs. pLXSN-flagHPV20E6 was constructed by cloning PCR amplified flag-tagged HPV20E6 into the pLXSN vector as previously described [41].

### Cell culture and transfection assays

H1299 cells (non-small cell lung carcinoma, p53-null, ATCC) were transfected with the respective HPV-E6 constructs as described previously [40]. Empty vector pcDNA3.1 (+) was used to equalize the total amount of transfected DNA in all samples and transfection efficiency was measured by co-expression of β-galactosidase (pCMV-β-gal). pLXSN without HPV20E6 was used as negative control in comparison with pLXSN-flag20E6.

### Retrovirus production and infection of NIKS cells

The spontaneously immortalized human foreskin keratinocyte cell line NIKS (Stratatech, Madison, USA) [42] was used for expression of HPV20E6. This cell line was maintained as sub-confluent cultures on a mitomycin C-treated fibroblast feeder layer in NIKS medium (3∶1 Ham's F-12 medium and Dulbecco's modified Eagle's medium (DMEM) supplemented with 5% FCS, 24 µg/adenine, 8,4 ng/ml cholera toxin, 10 ng/ml epidermal growth factor (EGF), 0,4 µg/ml hydrocortisone, 5 µg/ml insulin and 1% penicillin/streptomycin). NIKS cells were infected with pLXSN-flagHPVE6 retrovirus and pooled after selection with G418 as previously described [41]. The pooled cells (pLXSN and pLXSN-flag20E6) were subsequently kept on fresh mitomycin C-treated feeder layer in NIKS complete medium and expanded for 4 to 8 passages.

### Caspase- and ubiquitin-inhibitors

Transfected H1299 cells were incubated overnight with either 50 µM Z-VAD-FMK (general caspase inhibitor) or 25 µM calpeptin (calpain inhibitor) before harvesting. These transfected cells were alternatively incubated for 6 hours in the presence of the proteasome inhibitor MG132 (20 µM) or the 20S proteasome inhibitor β-lactone (Lactacystin 5 µM) (all Calbiochem, Darmstadt, Germany) before harvesting. HPV20E6-expressing NIKS cell line was incubated overnight with 10 µM MG132.

### Western blot analyses

Western blot was performed as described [40]. Total cellular protein was extracted and subjected to Western blot analysis after separation in 15% or 8% SDS-PAGE. For quantitation of the respective proteins, as well as to control for transfection efficiency, antibody staining for β-actin and β-galactosidase were respectively included in each analysis. The primary antibodies used were anti-Flag (cat#200472) (Stratagene, CA, USA), anti-p53 (sc-126), anti-p63α (sc-8344), anti-p16 (sc-9968) and anti-PCNA (sc-56) (all Santa Cruz, Heidelberg, Germany), anti-β-gal (cat#Z378A) (Promega, Madison, USA), anti-β-actin (cat#691001) (ICN, Aurora, Ohio, USA) and anti-pRB (cat#OP136) (Calbiochem, Darmstadt, Germany). Primary antibodies against Apaf-1 (cat#4452), caspase-3 (cat#9662), caspase-9 (cat#9509), caspase-8 (cat#9746), LC3B (cat#4108), Bak (cat#3792), Bax (cat#2774), phospho-c-Jun (ser63) (cat#9261) and c-Jun (cat#9165) were purchased from Cell Signaling (Frankfurt am Main, Germany).

### Apoptosis assays

Cells (with or without MG132 treatment) were collected by trypsinization, washed with phosphate-buffered saline and stained using an Annexin V-PE apoptosis Detection Kit I (BD Biosciences) as described previously [13]. Early stage apoptosis was identified as Annexin V positive(+)/7-AAD negative(−) population. Percentages were determined in the FACScalibur system using FlowJo software (Ashland, USA).

### Silencing of p53 by siRNA

siRNA p53 (10 nM; cat#1024849) and control siRNA which has no homology to any known mammalian gene (10 nM; cat#1022563) was transfected into the NIKS cell line as previously described [43]. Both reagents were purchased from Qiagen (Hilden, Germany). Control-siRNA and mock transfections (absence of siRNA) served as negative controls.

### Cell proliferation as measured by EdU incorporation

Cell proliferation was measured in three independent assays by EdU (5-ethynyl-2′-deoxyuridine) incorporation using the Click-iT™ EdU Flow Cytometry Assay Kit (Invitrogen, Karlsruhe, Germany) according to the manufacturer's protocol. Cells were treated with 10 µM EdU for 24 hours prior to staining with Alexa Fluor 488 azide (cat#C35002, Invitrogen, Karlsruhe, Germany). EdU incorporation was determined by FACScalibur using FlowJo software (Ashland, USA).

### Co-immunoprecipitation

Total protein was isolated from pLXSN-flagHPV20E6-NIKS cells as described previously [40] for co-immunoprecipitation assays. Lysates were pre-cleared to reduce background [44] and used for immunoprecipitation by adding 4 µg of the respective antibodies Cdk6, (Santa Cruz, Heidelberg, Germany), P16 (mtm, Heidelberg, Germany) and PP2AC (Cell Signal, Frankfurt am Main, Germany), followed by incubation for 1 h at 4°C on a rocking platform. Protein A-agarose (25 µl bed volume) was subsequently added to each immune complex and incubated overnight at 4°C. Reactions without cell lysates served as negative control. Immunocomplexes were washed according to the manufacturer's protocol (Roche, Mannheim Germany) before the immunoprecipitated proteins were dissociated from the beads by boiling for 3 min in one bed volume of elution buffer [126 mM Tris – HCl (pH 6), 20% glycerol, 4% SDS, 0.02% bromophenol blue, and 1% 2-mercaptoethanol]. The total sample was used for Western blots analysis [45]. Interaction partners with the immunoprecipitated proteins were visualized by Western blot analysis using the following respective antibodies Cdk6, Cdk4 (Santa Cruz, Heidelberg, Germany) CyclinD1, PP2AB, PP2AC (Cell Signaling, Frankfurt am Main, Germany) and P16 (mtm, Heidelberg, Germany).

### Methylation-specific PCR

Cellular DNA methylation was determined using the CpGenome™DNA Modification kit (Millipore, Schwalbach, Germany). Unmodified DNA was controlled for by amplification using W (wild type) primers. Methylation status of p16 CpG islands was analyzed by methylation-specific PCR amplification using the CpG WIZ® p16 Amplification Kit (Millipore, Schwalbach, Germany). Primers for amplification of methylated p16 CpG islands were (forward) 5′-TTA TTA GAG GGT GGG GCG GAT CGC-3′ and (reverse) 5′-GAC CCC GAA CCG CGA CCG TAA-3′ [46] and for unmethylated p16 CpG islands (forward) 5′-TTA TTA GAG GGT GGG GTG GAT TGT-3′ and (reverse) 5′-CAA CCC CAA ACC ACA AAC ATA A-3′. Genomic DNA of the colon cancer cell line RKO served as positive control in all reactions. PCR was performed with the HotStarTaq DNA polymerase kit (Qiagen, Hilden, Germany). Unmethylated p16 was amplified using the following conditions: 95°C for 5 min followed by 30 cycles of amplification (denaturation at 95°C for 30 s, annealing at 62°C (−0.3°C/cycle) for 30 s and elongation at 72°C for 30 s) plus 10 additional cycles of annealing at 53°C and a final extension at 72°C for 10 min [46]. Methylated p16 and wild type p16 were amplified in 45 cycles: Initial denaturation at 95°C for 12 min was followed by 10 cycles of denaturation at 94°C for 45 s, annealing at 68°C (methylated p16) or 60°C (wild type p16) for 45 s and elongation at 72°C for 45 s. This was followed by 35 cycles of denaturation at 94°C for 45 s, annealing at 63°C for methylated p16 or 58°C for wild type p16, elongation at 72°C for 45 s and additional a final extension step of 72°C for 10 min.

## Results

### p53 family-mediated stimulation or degradation of cutaneous HPV E6 depends on HPV type

**Figure 1:**
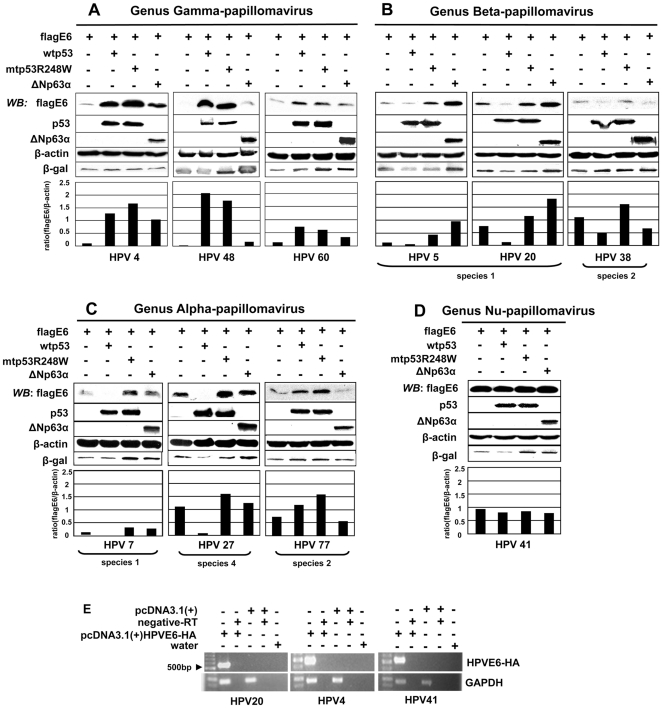
p53 family-mediated stimulation or degradation of cutaneous HPVE6 is HPV type dependent. Expression of HPVE6 genes in H1299 cells (p53-null) with co-expression of wtp53, mutp53R248W or ΔNp63α (1 µg each). HPV types included (A) HPV4E6, 48E6 and 60E6 of genus Gamma-papillomavirus, (B) HPV5E6, 20E6 and 38E6 from genus Beta-papillomavirus, (C) HPV7E6, 27E6 and 77E6 from genus Alpha-papillomavirus and (D) HPV41 from genus Nu-papillomavirus. Proteins were analysed by Western blot analyses and levels were quantified by Imagequant. Histograms represent HPV E6 protein expression normalized against β-actin loading control. Empty vector pcDNA3.1 (+) was co-transfected to equalize the total amount of transfected DNA. Transfection efficiency was controlled for by β-galactosidase expression. (E) RT-PCR [40], [59] demonstrating the respective mRNA of c-terminal HA-tagged HPVE6 genes.

We had previously demonstrated the wtp53 family-mediated degradation of HPV20E6 after ectopic expression of these proteins in the p53-null cell line H1299 [13]. In the present study we expressed N-terminal flag-tagged E6 proteins of a number of HPV types from different genera using the same conditions. These E6 proteins included HPV4, HPV48 and HPV60 of the genus Gamma-papillomavirus, from genus Beta-papillomavirus HPV5 and HPV20 (both species 1) and HPV38 (species 2), from genus Alpha-papillomavirus HPV7 (species 1), HPV77 (species 2) and HPV 27 (species 4), and HPV41E6 of genus Nu-papillomavirus. Proteins were extracted and Western blot analyses performed using antibodies against p53, ΔNp63α and flag for detection of flag-tagged E6 proteins (Figure 1). Transfection efficiency was measured by co-expression of β-galactosidase and the protein loading controlled with β-actin. Expression of both wtp53 and mutant p53R248W stimulated the expression of HPV4E6, HPV48E6 and HPV60E6 (genus Gamma-papillomavirus). Co-expression of HPV4E6 with ΔNp63α led to a higher stimulation of the viral E6 when compared to HPV48E6 and HPV60E6, but less than by p53 (Figure 1A).

The HPV types tested in genus Beta-papillomavirus reacted differently (Figure 1B). HPV20E6 expression was reduced in the presence of ectopically expressed wtp53, but protected by both mutp53R248W and ΔNp63α expression as previously reported [13]. This was similar when HPV5E6 was co-expressed with the respective wtp53, mutp53R248W and ΔNp63α. HPV38E6 in contrast, was down-regulated by co-expression of either wtp53 or ΔNp63α and stimulated by co-expression of mutp53R248W.

Differential results were also obtained for the HPV types belonging to genus Alpha-papillomavirus (Figure 1C). HPV7E6 and HPV27E6 were both down-regulated by co-expression with wtp53, whereas wtp53 expression increased the level of HPV77E6. Co-expression of mutp53R248W stimulated E6 expression of all three these HPV types, whereas ΔNp63α co-expression increased HPV7E6 and HPV27E6, but led to a decrease in HPV77E6.

Expression levels of HPV41E6 (genus Nu-papillomavirus) were hardly influenced by co-expression of any of the p53-family members (Figure 1D). C-terminal flag-tagged HPV20E6 was also tested. No difference was noted in comparison to the N-terminal flag-tagged HPV20E6 with respect to the down-regulation by wtp53, even though the obtained signal was weak (data not shown). C-terminal HA-tagged protein levels of all HPVE6 proteins could not be detected by Western blot analyses despite the demonstration of the respective mRNA production by RT-PCR (Figure 1E).

### Degradation of the respective HPV6 proteins

**Figure 2:**
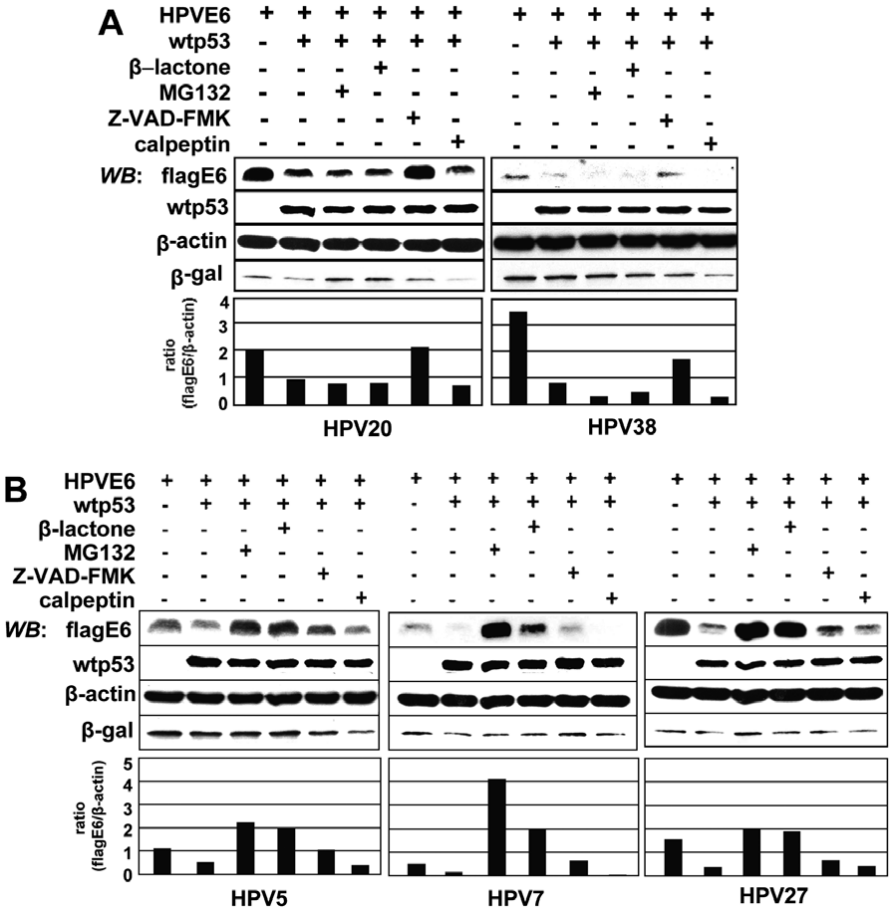
Degradation of the respective HPVE6 proteins. Wtp53 was co-expressed in H1299 cells with (A) HPV20E6 or HPV38E6, and (B) HPV5E6, HPV7E6 or HPV27E6. Transfected cells were treated with general caspase inhibitor Z-VAD-FMK (50 µM), calpeptin (calpain inhibitor, 25 µM) or proteasome inhibitors MG132 (20 µM) or β-lactone (Lactacystin 5 µM) prior to harvesting. Proteins were analysed by Western blot analyses and protein levels quantified by Imagequant. Histograms represent HPVE6 protein expression normalized against β-actin. Empty vector pcDNA3.1(+) was co-transfected to equalize total amount of transfected DNA and transfection efficiency was controlled for by β-galactosidase expression.

Caspase-3 is required for the degradation of HPV20E6 [13]. The degradation of the respective HPVE6 proteins in the presence of over-expressed wtp53 may be induced through different pathways. We therefore expressed the proteins in the p53-null H1299 cell line in the presence of either a general caspase inhibitor (Z-VAD-FMK), a calpain inhibitor (calpeptin) or proteasome inhibitors (MG132 or β-lactone), respectively (Figure 2). The reduction of HPV20E6 when co-expressed with wtp53, was eliminated in the presence of the caspase inhibitor Z-VAD-FMK. Calpeptin, MG132 or β-lactone in contrast, did not notably influence HPV20E6 levels. Similar observations were made when co-expressing HPV38E6 and wtp53 (Figure 2A). Treatment of H1299 transfected cells with proteasome inhibitors MG132 or β-lactone resulted in an increase of the respective HPV5E6, HPV7E6 or HPV27E6 protein levels in comparison to their levels under co-expression with wtp53 in untreated H1299 cells. Caspase-inhibitor Z-VAD-FMK seemed to rescue E6 levels of HPV5 and HPV7 from over-expressed wtp53, whereas the rescue in the case of HPV27E6 was very marginal. Calpeptin exerted a neutral or slightly negative effect (Figure 2B).

These data also indicate a HPV type difference in relation to the influence of cellular proteins involved in the degradation of the respective HPVE6 proteins. The effect is again independent of phylogenetic grouping.

### Influence of over-expressed wtp53, mtp53R248W and ΔNp63α on HPV20E6 in human keratinocytes harbouring endogenous wtp53

Degradation of HPV20E6 in the presence of ectopically expressed wtp53 in the p53-null H1299, a small cell lung cancer cell line, was used as model. This however raises the question whether similar observations can be made in human keratinocytes under the influence of endogenous wtp53 and p63. HPV20 does not readily immortalize primary keratinocytes. Difficulties were encountered in obtaining sufficient numbers of cultured primary keratinocytes expressing HPV20E6 for repeat experiments. We instead used the spontaneously immortalized human keratinocyte cell line NIKS [42] which also harbours endogenous p53-family members. Even though we demonstrated HPV20E6 mRNA by RT-PCR after transient transfection into NIKS cells (Figure S1), we failed to detect N-terminal flag-tagged HPV20E6 protein in these cells. We therefore established NIKS cells constitutively expressing N-terminal flag-tagged HPV20E6 using retroviral constructs (pLXSN-flagHPV20E6). Cells were pooled after G418 selection. We established and used 4 individual HPV20E6 expressing cell lines in order to cover any influences exerted by varying chromosomal integration loci. Similar cell lines were established for N-terminal flag-tagged HPV41E6, HPV27E6, HPV38E6 and HPV4E6, respectively. mRNA of the respective E6 genes was demonstrated in each case in these cells by RT-PCR, but only flagHPV20E6 and flagHPV41E6 proteins were detectable by Western blot analyses. We failed to detect expression of C-terminal HA-tagged E6 protein in pLXSN-HPV20E6-, -HPV4E6- or -HPV41E6-NIKS cells, respectively.

We restricted all further experiments in this study to the use of the 4 lines established for pLXSN-flagHPV20E6 for two reasons: detection of flagHPV20E6 in Western blot analyses and the differing influence of the p53 family members on its expression.

**Figure 3:**
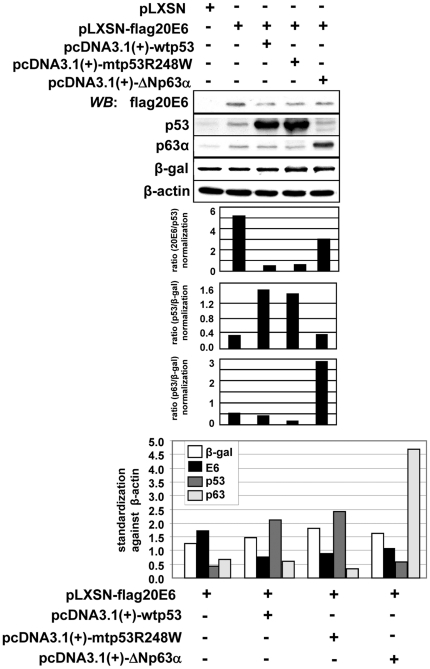
Influence of over-expressed wtp53, mtp53R248W and ΔNp63α on HPV20E6 expression in human keratinocytes with endogenous wtp53. HPV20E6 protein expression in pLXSN-flafHPV20E6-NIKS cells in the presence of wtp53, mtp53R248W or ΔNp63α (1 µg) as measured by Western blot analyses. Protein levels were quantified by Imagequant and histograms represent HPVE6 protein expression normalized against β-actin. Empty vector pcDNA3.1(+) was co-transfected to equalize total amount of transfected DNA and transfection efficiency was controlled for by β-galactosidase expression.

Equal amounts of wtp53-, mtp53R248W- and ΔNp63α-expressing constructs were transfected into the pLXSN-flagHPV20E6 cells. Expression levels of the respective proteins were measured in Western blot analyses. Co-transfection of β-galactosidase controlled for transfection efficiency. Protein expression was standardized against β-actin and normalized against β-galactosidase. Ectopically-expressed wtp53 and mutp53R248W both down-regulated HPV20E6 in the presence of endogenous p53 when compared to the flagHPV20E6 protein level (Figure 3). These results are contrary to observations made in H1299 cells in the absence of endogenous wtp53, where mutp53R248W had a protective effect [13]. A possible explanation is that tetramerization between endogenous wtp53 and mtp53R248W may lead to a gain of wtp53 function for the mutant p53 protein [26]. Similarly, ectopic expression of ΔNp63α at high levels in the keratinocytes also led to down-regulation of HPV20E6. Here tetramerization of endogenous p53 with ectopically expressed ΔNp63α may have a neutralizing effect on the latter.

### Other cellular factors also exert an influence on HPV20E6 protein expression

**Figure 4:**
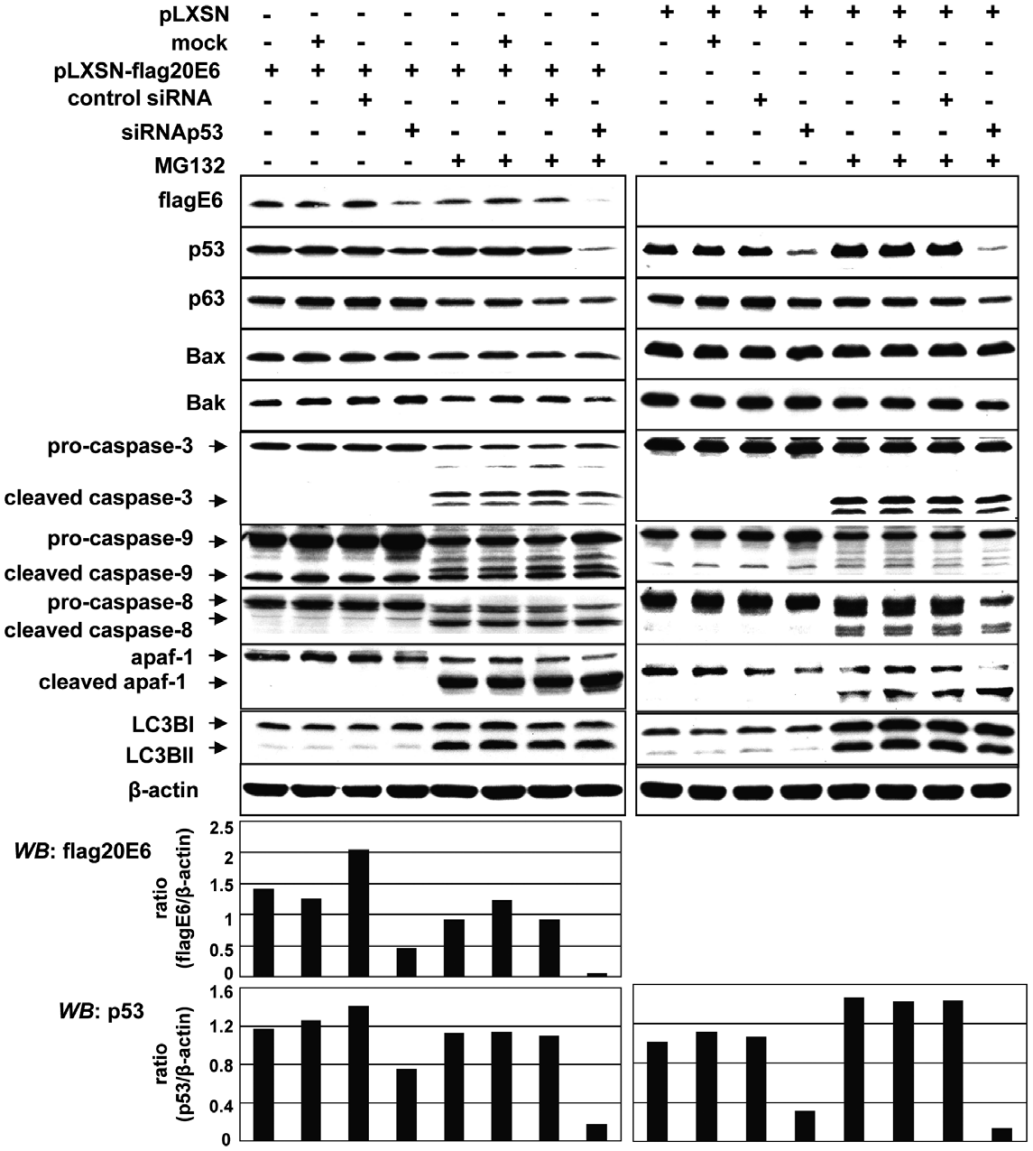
Down-regulation of HPV20E6 is influenced by proteins other than those involved in apoptosis or autophagy. Western blot analysis of cell lysates from pLXSN-flagHPV20E6- and pLXSN-NIKS cells after transfection with siRNAp53 (10 nM) or siRNA-NK (negative control siRNA, 10 nM) and incubation for 16 h with or without MG132 (10 µm). MG132 treatment induced activation of apaf-1, caspase-8, caspase-9 and caspase-3, as well as the autophagy marker LC3BII. Histograms represent HPV20E6 and wtp53 protein levels normalized against β-actin.

**Figure 5:**
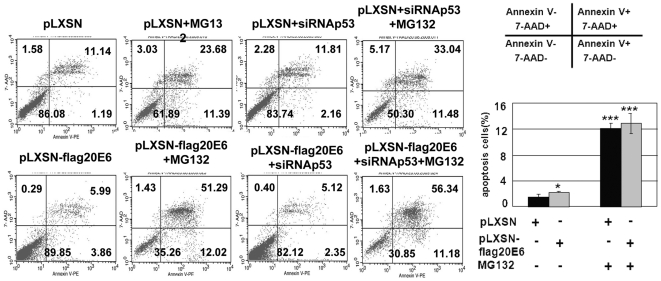
HPV20E6 expression itself does not induce apoptosis. pLXSN-flag20E6- or pLXSN- NIKS cells were incubated overnight with MG132 (10 µM). Apoptosis was subsequently measured by flow cytometry of Annexin V-staining cells (Annexin V+/7-AAD-) in three independent experiments. Apoptosis [Annexin V(+)/7-AAD(−)] was significantly increased in both pLXSN20E6- and pLXSN-NIKS cells after MG132 treatment (p<0,001), but no significant difference (p = 0,042) was seen between HPV20E6-expressing cells in comparison to controls. Statistical analyses were performed using the student t-test. * - p<0.05, *** - p<0.001.

Endogenous p53 was partially silenced by transfecting siRNAp53 into pLXSN-flagHPV20E6 cells. Total elimination of p53 by siRNA is not possible under experimental conditions [47]. Surprisingly, the level of HPV20E6 was concomitantly down-regulated in the presence of siRNAp53 in contrast to the negative controls (untransduced, transfected with control-siRNA or mock transfected cells) (Figure 4). These data indicate that cellular factor(s) other than endogenous p53, may be involved in the degradation of HPV20E6. Transduced cells were then treated with MG132 to determine whether proteasomal degradation was involved. This led to a further decrease of HPV20E6 protein after transfection with siRNAp53 in comparison to down-regulation in untreated cell lines. Results point to a sensitivity of this unknown cellular factor to proteasomal degradation. Interestingly, MG132 treatment alone did not influence endogenous p53 levels, whereas silencing of the latter by siRNAp53 was strongly augmented under MG132 treatment and independent of expression of HPV20E6 (Figure 4). We investigated whether other cellular proteins known to be involved in protein degradation, may be involved in the down-regulation of HPV20E6 and endogenous p53 in pLXSN-flagHPV20E6 and control pLXSN cells in the presence or absence of MG132 (Figure 4). Apaf-1, caspase-3, caspase-9 and caspase-8 were all activated in the presence of MG132 as measured by their respective cleaved proteins, indicating activation of both extrinsic and intrinsic apoptotic pathway in both cell lines. Similarly, LC3BI was activated to LC3BII indicating involvement of autophagy [48]. Activation of these proteins in the presence of MG132 has previously been reported [49], [50]. The HPV20E6 and wtp53 levels were minimally reduced under these conditions. Interestingly, wtp53 expression in MG132 treated cells was decreased in the presence of HPV20E6 expression when compared to control cells. We subsequently performed FACS analyses in order to determine whether an apoptotic effect of these proteins could lead to the reduction in HV20E6 protein (Figure 5). The very low rate of apoptosis (range of 2%) measured in both cell lines, with a slightly higher rate in pLXSN-flagHPV20E6 cells in comparison to pLXSN cells (p<0.05), was significantly increased in both cell lines after MG132 treatment (p<0.001). The presence of siRNAp53 did not influence this apoptosis. Molecular regulators of apoptosis and autophagy are inter-connected and can be activated by a variety of death stimuli [51]. These results indicate that the mere activation of pathways involved in apoptosis or autophagy did not result in the reduced HPV20E6 and endogenous p53 protein levels.

Previous studies reported that cutaneous HPVE6 prevented apoptosis after UV exposure by inducing degradation of the pro-apoptotic Bak protein and preventing release of AIF from mitochondria [37], [38]. Constitutive Bak was however not degraded in the presence of HPVE6 [37]. In the present study neither Bak nor Bax was degraded in the presence of HPV20E6 (Figure 4).

### HPV20E6 expression leads to increased proliferation of keratinocytes

**Figure 6:**
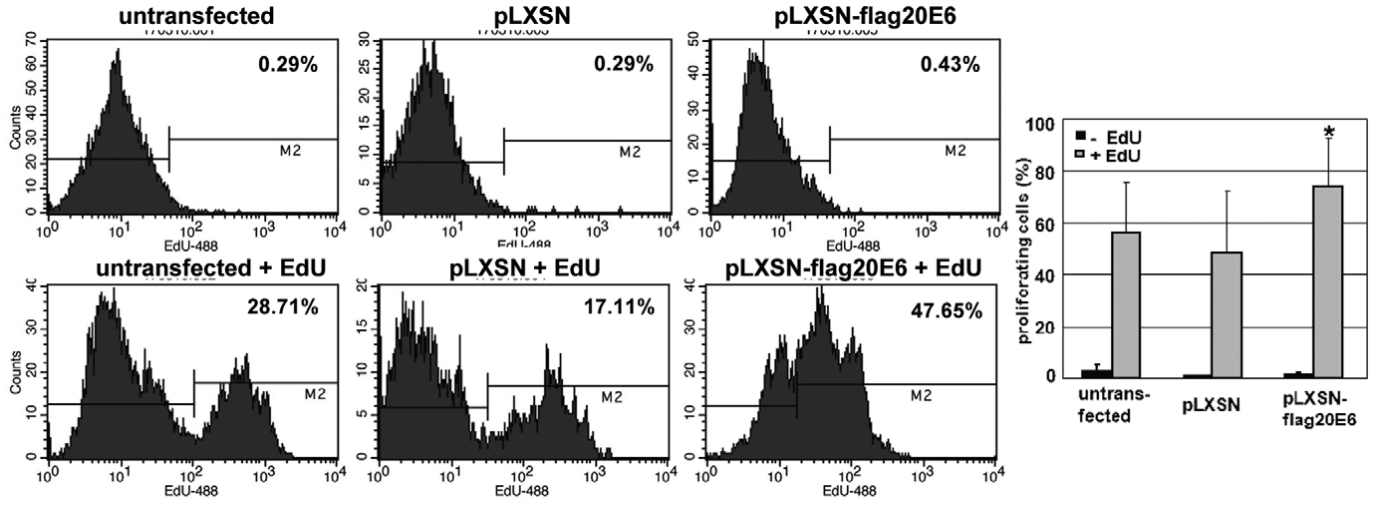
HPV20E6 expression increases proliferation. Equal densities of pLXSN-20E6 and pLXSN cells were incubated with 10 µM EdU for 24 hours. EdU-7AAD staining was analysed by FACS for the percentage of proliferating cells as indicated in the histogram. Statistical analyses (student t-test) of three independent experiments indicate a significant increase in the proliferation rate of pLXSN-20E6 cells (*p<0.05) in comparison to pLXSN cells and untransduced NIKS cells.

As the low levels of apoptosis did not markedly influence growth of the pLXSN-flagHPV20E6 cell line, we continued to explore whether even low level HPV20E6 expression provides a proliferative advantage to keratinocytes. EdU incorporation into the cells was used to measure proliferation (Figure 6). Increased proliferation rate of pLXSN-flagHPV20E6 cells was significantly higher than in the pLXSN control cells (p<0.05) or untransfected NIKS cells (p<0.05).

These results prompted us to investigate whether HPV20E6 expression influenced cellular proteins involved in cell cycle regulation.

### HPV20E6 does not influence interaction between cyclin-D1 and cdk6

**Figure 7:**
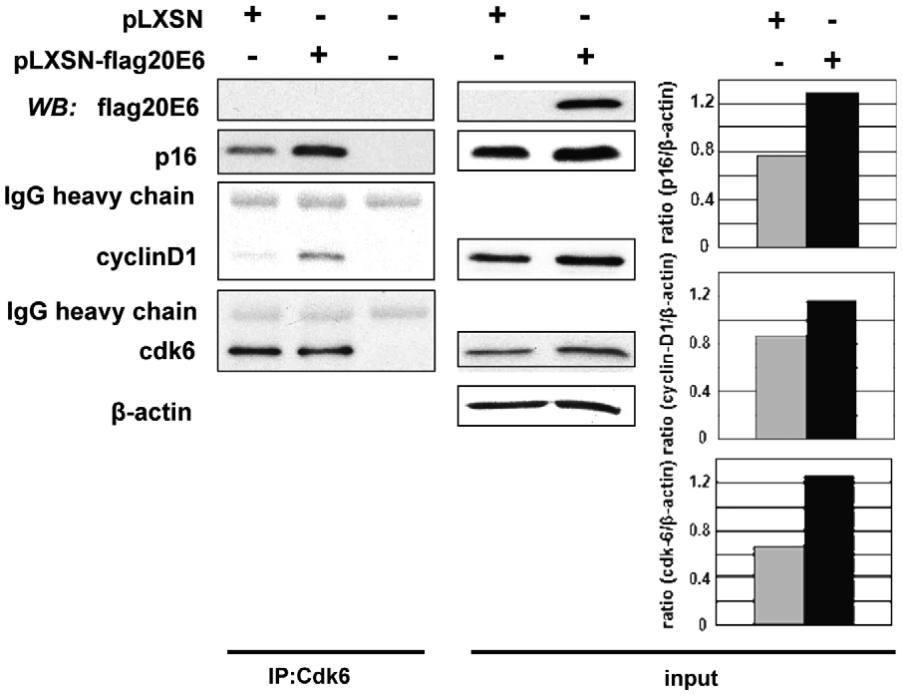
HPV20 E6 does not compete for binding of cdk6 to cyclinD1 or to p16^INK4a^. Total protein extracts from pLXSN-flag20E6- or pLXSN-NIKS cells were immuno-precipitated onto cdk6 protein. Protein A agarose precipitated with cdk6 antibody alone served as negative control. Levels of input proteins flagHPV20E6, p16, cyclinD1 and cdk6 were visualized by western blot analyses. Histograms represent input protein expression normalized to β-actin serving as loading control.

An important step in cell cycle control is the complex formation between cyclin-D1 and the cyclin-dependent kinases cdk4/6 to drive cells from the early to the late G1 phase of the cell cycle. The INK4 family of cyclin-dependent kinase inhibitors (including p16^INK4a^) dysregulates this step by binding to and inactivating cyclinD1-cdk4/6 complexes [52]. We investigated whether HPV20E6 may contribute to cell proliferation by interacting with cyclin-D1, cdk6 and/or p16^INK4a^. We performed competitive co-immunoprecipitation assays between HPV20E6 and these cellular proteins. Cyclin-D1 and p16^INK4a^ were pulled down by cdk6 antibodies without involvement of HPV20E6 (Figure 7). Interestingly, expression of HPV20E6 increased the protein levels of cyclin-D1, cdk6 as well as p16^INK4a^ in comparison to the control pLXSN cells. Inverse co-immunoprecipitation assays excluded non-specific interactions. Only unmethylated p16 was present in both pLXSN-flagHPV20E6 and pLXSN cell lines as demonstrated by methylation specific PCR analyses (Figure S2).

### PP2A does not play a role in HPV20E6-induced keratinocyte proliferation

PP2A is a major serine-phosphatase. SV40 small t-antigen binds to the A subunit of PP2A by displacing its B subunit. This binding induces upregulation of cyclin-D1 transcription which leads to cell transformation [53], [54]. We investigated whether HPV20E6 is able to interact with PP2A in a similar way by performing co-immunoprecipitation assays. We failed to demonstrate binding between HPV20E6 with either the AC subunits or the B subunit of PP2A (data not shown).

c-Jun enhances cell proliferation through the induction of cyclin-D1 transcription, whereas PP2A represses AP-1 activity by dephosphorylation of c-Jun on Ser63 [55]. We therefore also determined the levels of both phosphorylated and un-phosphorylated c-Jun in the pLXSN-flagHPV20E6 cells. The levels of both forms of c-Jun were increased in the HPV20E6 expressing cells in comparison to the pLXSN control cells (Figure S3).

### pRB is phosphorylated in the presence of HPV20E6 expression

**Figure 8:**
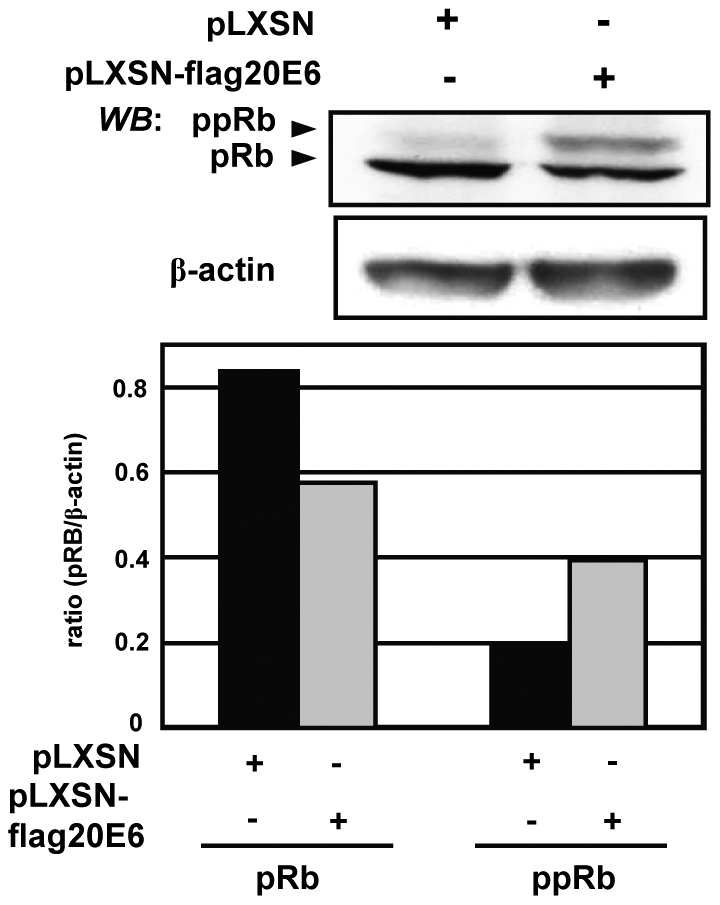
HPV20E6 increases the expression of ppRB. Western blot analyses of the protein level of pRb and ppRb in pLXSN-flag20E6 and pLXSN transduced NIKS cells. The pRb and ppRb protein level were quantified by Imagequant. Histograms were adjusted for β-actin which was used as a loading control.

The inactivation of pRB leads to suppression of senescence allowing the cell to maintain the proliferative state by entry into S-phase [56]. Phosphorylation of pRB was increased in the presence of HPV20E6 expression, as demonstrated by western blot analyses (Figure 8), indicating inactivation of this protein.

## Discussion

HPV infection, UV-exposure and mutations in wtp53 have been considered as interacting factors in the pathogenesis of non-melanoma skin cancer. DNA of a wide spectrum of HPV types, mainly of the genera Beta- and Gamma-papillomaviruses has been demonstrated not only in the malignant lesions, but also in normal skin [3], [4]. Recent data failed to demonstrate HPV transcription in these lesions thereby posing the question whether these viruses are actually involved in the pathogenesis of this disease [57]. We have previously demonstrated that the activation or suppression of a number of cutaneous HPV promoters by UV-irradiation is type specific rather than genus- or species-specific [39]. This was recently confirmed by others [36]. Chronic exposure of HPV20- and HPV27E6/E7 transgenic mice to UV-irradiation resulted in the formation of papillomas and malignant lesions of the skin [58]. Our previous studies demonstrated interplay between UV-induced mutp53R248W, ΔNp63α and wtp53 in relation to the expression of HPV20E6. Wtp53 mediated the caspase-3-dependent degradation of HPV20E6 protein [13], [40], [59]. We now extended these investigations to other HPV types of the genera Beta-, Gamma-and Nu-papillomavirus. Co-expression with wtp53 exerted a very distinct regulation for the individual cutaneous E6 genes. E6 proteins of all types from genus Gamma-papillomavirus tested (HPV4, HPV48 and HPV 60) were up-regulated by wtp53, in contrast to the E6 down-regulation of all Beta-papillomavirus types tested (HPV5, HPV20 and HPV38). Wtp53 down-regulated HPV7E6 and HPV27E6, but up-regulated HPV77E6, all from genus-Alpha-papillomavirus. Mutp53R248W up-regulated expression of all HPVE6 proteins tested. HPV38E6 and HPV77E6 were down-regulated by ΔNp63α whereas all other E6 proteins were up-regulated. Down-regulation of the respective HPV E6 proteins was type dependent and mediated either through caspase- and/or proteasome pathways. HPV41E6 expression remained unaffected by any of these p53-family members. These data underline the diversity in intracellular mechanisms exerted by various papillomavirus types, differing not only between genera, but even between HPV types within a genus.

We had obtained these results by over-expressing the respective proteins in a p53-null cell line. We now attempted to mimic the *in vivo* situation as close as possible by repeating these experiments using a spontaneously immortalized keratinocyte cell line (NIKS) harbouring endogenous p53 and p63. Ectopic expression of both mutp53R248W and ΔNp63α led to a down-regulation of HPV20E6 in contrast to our previous data obtained in p53-null cells, as well as in untransfected pLXSN-flagHPV20E6 cells. The contact-mutant p53R248W acts as dominant negative after tetramerization with wtp53. This complex re-localizes to the nucleus. p63 localizes predominantly to the nucleus in the presence of wtp53, but forms perinuclear aggregates when tetramerizing with mutant p53R248W [26]. Re-localization of these complexes may lead to altered function. An imbalance between endogenous p53 and p63 in relation to over-expressed mutp53R248W or ΔNp63α as given in our *in vitro* system may have influenced the functions of these proteins.

Endogenous p53 was partially silenced by transfecting siRNAp53 into pLXSN-flagHPV20E6 and pLXSN cells. Surprisingly this resulted in a reduced expression of HPV20E6 as well. MG132 treatment of these cells led to the expected activation of several cellular proteins involved in both apoptotic and autophagic pathways, but did not alleviate the down-regulation of HPV20E6. Partial silencing of p53 by addition of siRNAp53 did not influence the increased apoptosis in MG132-treated pLXSN-flagHPV20E6 and pLXSN cells as measured by FACS analyses. We therefore conclude that HPV20E6 expression is modulated by additional, yet unidentified, cellular protein(s) which are not necessarily involved in apoptosis or autophagy.

HPV20E6 expression induced proliferation of the pLXSN-flagHPV20E6 cells. In an attempt to identify cellular proteins involved in cell cycle control which may be affected by the expression of HPV20E6, we investigated cyclin-D1, cdk6, p16^INK4a^, PP2A and pRB. Cyclin-D1 and ckd6 enhance keratinocyte proliferation by phosphorylation of down-stream targets including pRB [60], [61]. The increased pLXSN-flagHPV20E6 proliferation may be a consequence of increased expression of cell cycle proteins indirectly induced by HPV20E6 expression. However, p16^INK4a^ is active and its expression elevated in our system. We demonstrated binding of p16^INK4a^ to the CDK complexes which would negatively regulate proliferation. HPV20E6 did not compete for this interaction of p16^INK4a^ with cyclin-D1 or cdk6. p16^INK4a^ similarly activates pRB by preventing its phosphorylation. However, the level of pRB phosphorylation in the HPV20E6 expressing cells would be sufficient to override the cytokenetic block induced by the p16^INK4a^/pRB pathway [62].

HPV16E7 binds to subunits of PP2A thereby sequestering de-phosphorylation of down-stream targets involved in proliferation [63]. PP2A does not seem to be directly involved in our system. HPV20E6 does not bind either A or B subunits and PP2A in turn does not influence the phosphorylation of c-Jun which is necessary for the activation of cyclin-D1 transcription. The levels of both p-c-Jun and c-Jun were elevated in the HPV20E6 expressing cells.

The p53 and p63 proteins (including p53 mutants) are involved in a plephora of cellular functions [24], [26]–[28], [64]. Many of these functions are attributed to complex interactions between p53 and p63 isoforms and their mutants, which in turn influence the activity of downstream targets involved in development and proliferation. A delicate balance between protein levels of individual cellular factors is very important and ratios between proteins often determine biological outcome [27]. The present study demonstrates the diverse influence of p53 family members on individual cutaneous HPVE6 proteins. HPV20E6 expression also resulted in varying protein levels of factors involved in proliferation and differentiation. Additional investigation for each cutaneous HPV type is needed to determine the exact role of individual viral proteins in relation to cellular factors involved in proliferation and differentiation. These observations underline the diverse clinical manifestations induced by individual cutaneous HPV types.

## Supporting Information

Figure S1 **RT-PCR demonstrating expression of HPVE6.** N-terminal-flag-HPVE6 of the respective HPV types was transiently transfected into NIKS cells. Total RNA was subsequently isolated and used for RT-PCR. GAPDH served as internal control.
(TIF)

Figure S2 **HPV20E6 influenced neither the methylated nor unmethylated p16.** Methylation status of the bisulfied p16 CpG islands was analysed by PCR amplification using primers specific for methylated or unmethylated p16. RKO was used as positive control of methylated p16. W (wild type) primers amplify only DNA which is not chemically modified and serve as a control for the efficiency of chemical modification. Gene expression was measured from three independent experiments and histograms represent unmethylated p16 normalized against wild type p16. No significant difference (student t-test) was determined between pLXSN-flag20E6 NIKS cells and the respective controls: (1) untransfected NIKS cells (2) pLXSN-NIKS cells (3) pLXSN-flag20E6-NIKS cells and (4) RKO cells.
(TIF)

Figure S3 **HPV 20E6 up-regulated c-Jun and p-c-Jun protein levels.** c-Jun and p-c-Jun levels in pLXSN-flag20E6 and pLXSN-NIKS cells by Western blot analyses. Histograms indicate levels adjusted against β-actin which served as loading control.
(TIF)

Table S1 **Primers for plasmid design.** Primers used for PCR amplification of N-terminal or C-terminal flag-tagged E6 and C-terminal hemagglutinin (HA)-tagged E6. Full-length genomes were used as template for the E6 amplification of HPV types 4, 5, 7, 20, 27, 38, 41, 48, 60 and 77.
(DOC)
