# Supplementary figures and images for: Differential Regulation of Cutaneous Oncoprotein HPVE6 by wtp53, Mutant p53R248W and ΔNp63α is HPV Type Dependent
Source: PLoS One. 2012 Apr 18;7(4):e35540. doi: 10.1371/journal.pone.0035540 (PMC3329482; doi:10.1371/journal.pone.0035540)

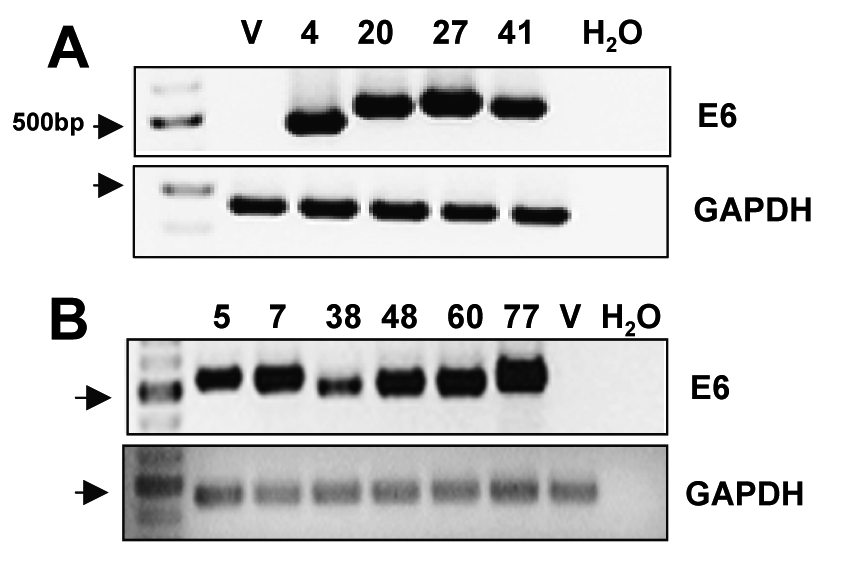

Supplement: Figure S1 — RT-PCR demonstrating expression of HPVE6. N-terminal-flag-HPVE6 of the respective HPV types was transiently transfected into NIKS cells. Total RNA was subsequently isolated and used for RT-PCR. GAPDH served as internal control. (TIF) [file pone.0035540.s001.tif]

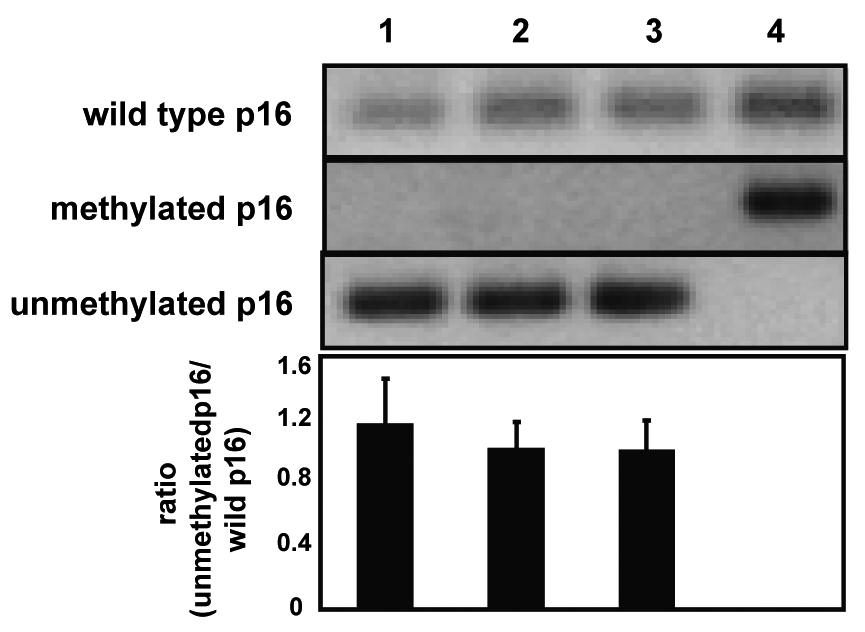

Supplement: Figure S2 — HPV20E6 influenced neither the methylated nor unmethylated p16. Methylation status of the bisulfied p16 CpG islands was analysed by PCR amplification using primers specific for methylated or unmethylated p16. RKO was used as positive control of methylated p16. W (wild type) primers amplify only DNA which is not chemically modified and serve as a control for the efficiency of chemical modification. Gene expression was measured from three independent experiments and histograms represent unmethylated p16 normalized against wild type p16. No significant difference (student t-test) was determined between pLXSN-flag20E6 NIKS cells and the respective controls: (1) untransfected NIKS cells (2) pLXSN-NIKS cells (3) pLXSN-flag20E6-NIKS cells and (4) RKO cells. (TIF) [file pone.0035540.s002.tif]

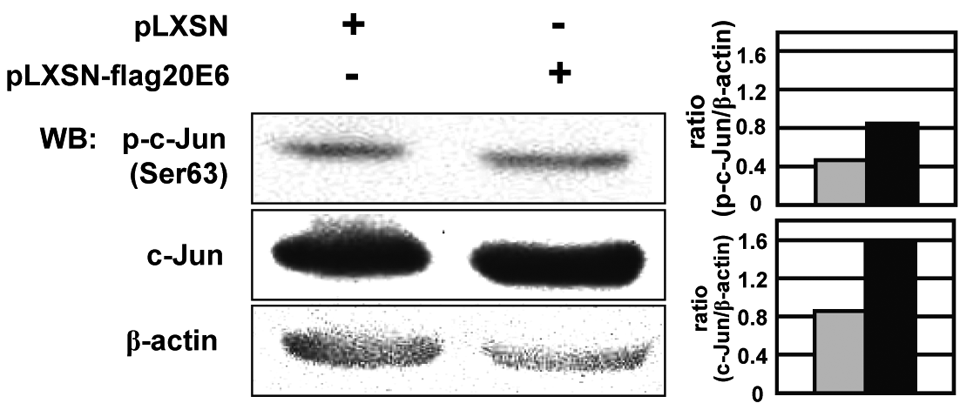

Supplement: Figure S3 — HPV 20E6 up-regulated c-Jun and p-c-Jun protein levels. c-Jun and p-c-Jun levels in pLXSN-flag20E6 and pLXSN-NIKS cells by Western blot analyses. Histograms indicate levels adjusted against β-actin which served as loading control. (TIF) [file pone.0035540.s003.tif]
