# Supplementary material for: Differential Regulation of Cutaneous Oncoprotein HPVE6 by wtp53, Mutant p53R248W and ΔNp63α is HPV Type Dependent
Source: PLoS One. 2012 Apr 18;7(4):e35540. doi: 10.1371/journal.pone.0035540 (PMC3329482; doi:10.1371/journal.pone.0035540)
Supplement: Table S1 — Primers for plasmid design. Primers used for PCR amplification of N-terminal or C-terminal flag-tagged E6 and C-terminal hemagglutinin (HA)-tagged E6. Full-length genomes were used as template for the E6 amplification of HPV types 4, 5, 7, 20, 27, 38, 41, 48, 60 and 77. (DOC) [file pone.0035540.s004.doc]

**Table S1.**

| **HPV**  **type** | **N-terminal flag-tagged E6** |
| --- | --- |
| 4E6f | 5’-CGGGATCCCGACCATGGACTACAAGGACGACGACGACAAGG  CAGATGGCAG-3’ |
| 4E6r | 5’-GCTCTAGAGCTCATTGTTTCCTAATACAATTTC-3’ |
| 5E6f | 5’-CGGGATCCCGACCATGGACTACAAGGACGACGACGACAAGGCT  GAGGGAGC-3’ |
| 5E6r | 5’-GCTCTAGAGCTTACCAATCATGATAAAAATGC-3’ |
| 7E6f | 5’-CGGGATCCCGACCATGGACTACAAGGACGACGACGACAAGTCT  GCACGTTG-3’ |
| 7E6r | 5’-GCTCTAGAGCCTAACACGATGTCTCCGAGCG -3’ |
| 20E6f | 5’- AAGGATCCACCATGGACTACAAGGACGACGACGACAAGGCTA  CACCTC-3’ |
| 20E6r | 5’-GCTCTAGAGCTTATTGAAAATGCTTACACAGCCTACGAGTTCC  TTTCCAAGAGCCTCTC-3’ |
| 27E6f | 5’-CGGGATCCCGACCATGGACTACAAGGACGACGACGACAAGCG  CACAAGGGC-3’ |
| 27E6r | 5’-GCTCTAGAGCTTAATGTAATGTCCGCGAGGC-3’ |
| 38E6f | 5’-CGGGATCCCGACCATGGACTACAAGGACGACGACGACAAGG  AACTACCAAAACC-3’ |
| 38E6r | 5’-GCTCTAGAGCTCATTCTATTGCTTTGCAATGCC-3’ |
| 41E6f | 5’-CTAGCTAGCTAGACCATGGACTACAAGGACGACGACGACAAGG  CATCAACAAGC-3’ |
| 41E6r | 5’-GCTCTAGAGCCTAATACAGTCCTACCATACATCG-3’ |
| 48E6f | 5’-CGGGATCCCGACCATGGACTACAAGGACGACGACGACAAGGAG  CCACAA-3’ |
| 48E6r | 5’-GCTCTAGAGCTTATCTCCCCTCATGGCTGATAC -3’ |
| 60E6f | 5’-CGGGATCCCGACCATGGACTACAAGGACGACGACGACAAGGA  AGAAGACAGG-3’ |
| 60E6r | 5’-GCTCTAGAGCTCATTTCTCAATGCAATTTCTAC -3’ |
| 77E6f | 5’-CGGGATCCCGACCATGGACTACAAGGACGACGACGACAAGTCT  ACAAGTG-3’ |
| 77E6r | 5’-GCTCTAGAGCTTACTGTCGGCTTTGGCC-3’ |
|  | **C-terminal flag-tagged E6** |
| 20E6f | 5’- GGGGATCCCCATGGCTACACCTCCTTCTTCAGAAG-3’ |
| 20E6r | 5’-GCTCTAGAGCTTACTTGTCGTCGTCGTCCTTGTAGTCTTGAAAA  TGCTTACACAGCC-3’ |
|  | **C-terminal HA-tagged E6** |
| 4E6f | 5’- CGGAATTCCGACCATGGCAGATGGCAGACCTGC-3’ |
| 4E6r | 5’-CCGCTCGAGCGGTCAAGCGTAGTCTGGGACGTCGTATGGGTA  TTGTTTCCTAATACAATT TC-3’ |
| 20E6f | 5’-CGGAATTCCG ACCATGGCTACACCTCCTTCTTCAG-3’ |
| 20E6r | 5’-CCGCTCGAGCGGTTAAGCGTAGTCTGGGACGTCGTATGGGTAT  TGAAAATGCTTACACAG-3’ |
| 41E6f | 5’-CGGAATTCCGACCATGGCATCAACAAGCGGTGTGGGA-3’ |
| 41E6r | 5’-CCGCTCGAGCGGCTAAGCGTAGTCTGGGACGTCGTATGGGTAATA  CAGTCCTACCATACATCGAACAC-3’ |

f – forward r - reverse
